# Supplementary material for: Breaking the fast: first report of dives and ingestion events in molting southern elephant seals
Source: Commun Biol. 2024 Jan 8;7:64. doi: 10.1038/s42003-023-05720-2 (PMC10774426; doi:10.1038/s42003-023-05720-2)
Supplement: Supplementary file 2 — Supplementary Information [file 42003_2023_5720_MOESM2_ESM.pdf]

Supplementary information

Breaking the fast: First report of dives and ingestions events in moulting southern elephant seals.

Laura Charlanne[1], Laureline Chaise[2], Damien Sornette[2], Erwan Piot[3,4], Dominic J. McCafferty[5], André Ancel[1], Caroline Gilbert[4,6].

[1]Université de Strasbourg, CNRS, IPHC UMR 7178, F-67000 Strasbourg, France

[2]Hex·Data, 847 Route de Frans, 69400 Villefranche-sur-Saône, France (https://hex-data.io)

[3] CNRS UMR5536, Université de Bordeaux, 33076 Bordeaux, France

[4]UMR 7179, CNRS/MNHN, Laboratoire MECADEV, 1 avenue du petit château, 91400, Brunoy, France

[5]Scottish Centre for Ecology and the Natural Environment, School of Biodiversity, One Health and Veterinary Medicine, College of Medical Veterinary and Life Sciences, University of Glasgow, Glasgow, UK

[6]Ecole Nationale Vétérinaire d’Alfort, 7 avenue du Général de Gaulle, 94704 Maisons-Alfort, France

Supplementary Figure S1. At-sea movement recorded in one monitored female southern elephant seal at mid-stage of moult in 2016, from the site of Pointe Suzanne (49°26'S, 70°26'E), with a corresponding maximum dive depth of 18.5 m. Red arrows represent the direction of travel between the grey dots (Argos locations).

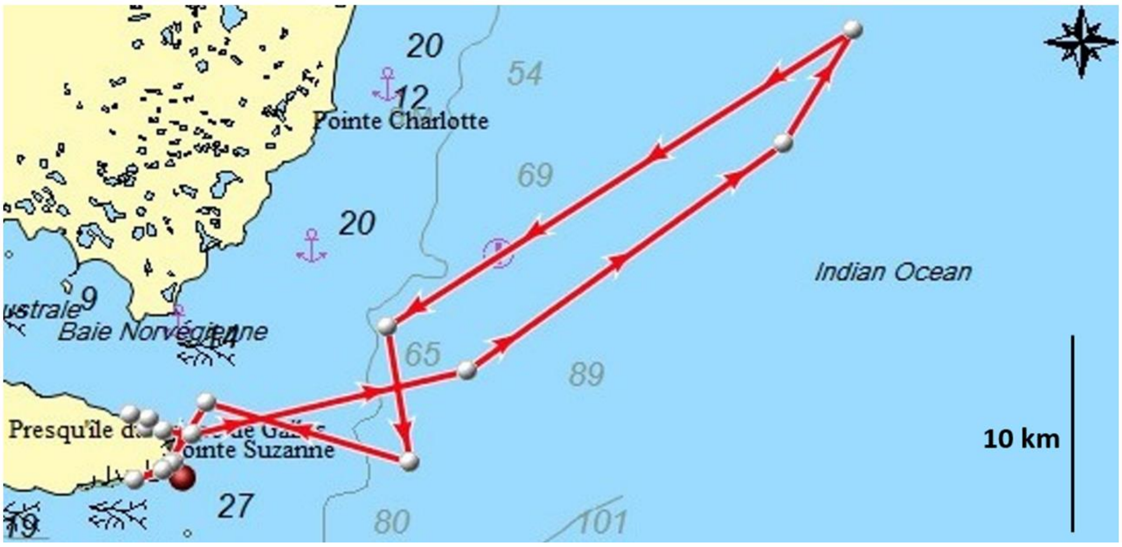

Supplementary Figure S2. Method used to calculate the area above the curve of drop in stomach temperature during ingestion events.

We used the equation:

$$Area_{event} = |area_{trapezoid}| - (|T_3 - T_1| * (t_3 - t_1)) / 2$$

Where $area_{trapezoid}$ was calculated using the integral function "trapezoid()" from Python package "scipy" (version: 1.9.1), and T_1, T_3 (°C) and t_1, t_3 (s) are respectively the stomach temperature and time at points T_1 and T_3 (start and end of the ingestion event).

Recovery temperature T_3 can be greater than initial temperature T_1 (A) or lower (B) (based on Kuhn and Costa, 2006): the grey area below corresponds to $area_{trapezoid}$ calculated with the trapezoid method, and hatched area corresponds to half of the additional rectangular area calculated ($|T_3 - T_1| * (t_3 - t_1) / 2$).

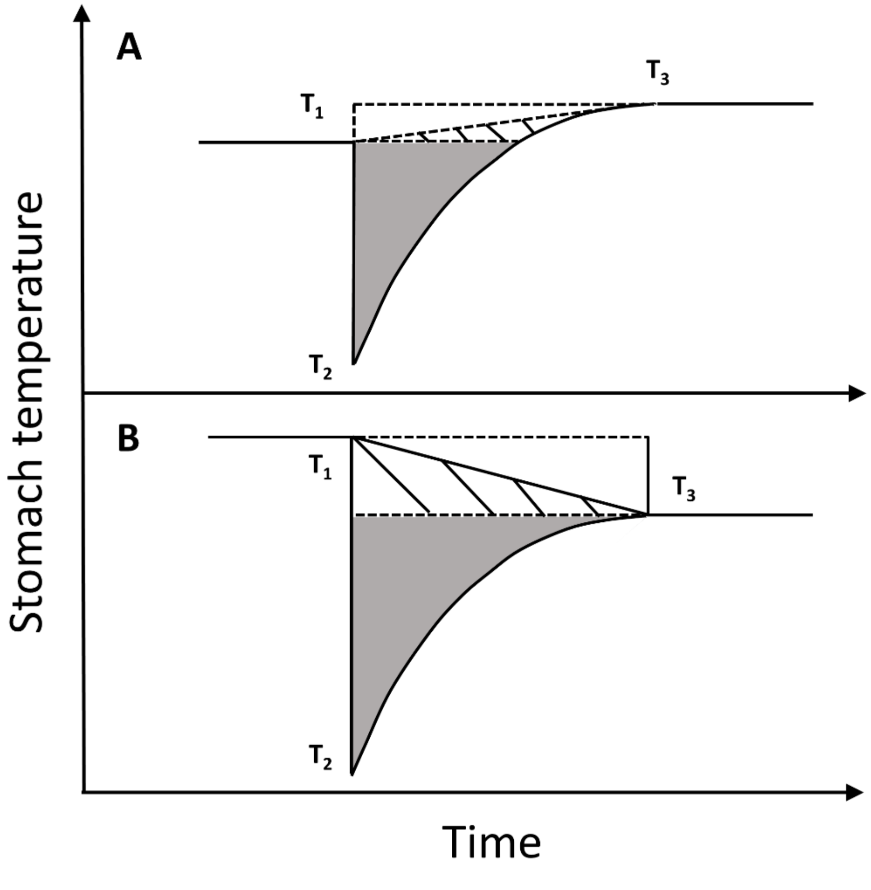

Supplementary Table S1: Summary of ingestion event variables from 31 female Southern elephant seals monitored between 2014 and 2022 (n = 87 events).

	Mean (SD)	Min - Max
Minimum temperature T_2 (°C)	33.8 (2.0)	27.1 - 36.5
Delta of temperature T_1- T_2 (°C)	3.4 (2.0)	0.5 - 10.7
Time to minimum temperature T_1- T_2 (min)	11.3 (30.4)	0.2 – 182.2
Time to temperature recovery T_2-T_3 (min)	36.9 (17.6)	9.2-– 98.2
Area above the curve (s.°C)	2361 (1536)	151 - 6976
Index of rate of temperature recovery I (s.°C^{-1})	474 (840)	13 - 5213
