# Supplementary material for: Breaking the fast: first report of dives and ingestion events in molting southern elephant seals
Source: Commun Biol. 2024 Jan 8;7:64. doi: 10.1038/s42003-023-05720-2 (PMC10774426; doi:10.1038/s42003-023-05720-2)
Supplement: Supplementary file 3 — Reporting Summary [file 42003_2023_5720_MOESM3_ESM.pdf]

nature portfolio

Corresponding author(s): Laura Charlanne

Last updated by author(s): Dec 11, 2023

Reporting Summary

Nature Portfolio wishes to improve the reproducibility of the work that we publish. This form provides structure for consistency and transparency in reporting. For further information on Nature Portfolio policies, see our Editorial Policies and the Editorial Policy Checklist.

Statistics

For all statistical analyses, confirm that the following items are present in the figure legend, table legend, main text, or Methods section.

n/a	Confirmed	
☐	☒	The exact sample size (n) for each experimental group/condition, given as a discrete number and unit of measurement
☐	☒	A statement on whether measurements were taken from distinct samples or whether the same sample was measured repeatedly
☐	☒	The statistical test(s) used AND whether they are one- or two-sided<br>*Only common tests should be described solely by name; describe more complex techniques in the Methods section.*
☐	☒	A description of all covariates tested
☐	☒	A description of any assumptions or corrections, such as tests of normality and adjustment for multiple comparisons
☐	☒	A full description of the statistical parameters including central tendency (e.g. means) or other basic estimates (e.g. regression coefficient) AND variation (e.g. standard deviation) or associated estimates of uncertainty (e.g. confidence intervals)
☐	☒	For null hypothesis testing, the test statistic (e.g. F, t, r) with confidence intervals, effect sizes, degrees of freedom and P value noted<br>*Give P values as exact values whenever suitable.*
☒	☐	For Bayesian analysis, information on the choice of priors and Markov chain Monte Carlo settings
☒	☐	For hierarchical and complex designs, identification of the appropriate level for tests and full reporting of outcomes
☒	☐	Estimates of effect sizes (e.g. Cohen's d, Pearson's r), indicating how they were calculated

Our web collection on statistics for biologists contains articles on many of the points above.

Software and code

Policy information about availability of computer code

Data collection	Data were collected directly in the field
Data analysis	A Python algorithm was designed for the study by Hex Data, France (Python 3.10, "pandas" 1.5). We used R software for other statistics (4.1.2)

For manuscripts utilizing custom algorithms or software that are central to the research but not yet described in published literature, software must be made available to editors and reviewers. We strongly encourage code deposition in a community repository (e.g. GitHub). See the Nature Portfolio guidelines for submitting code & software for further information.

Data

Policy information about availability of data

All manuscripts must include a data availability statement. This statement should provide the following information, where applicable:

- Accession codes, unique identifiers, or web links for publicly available datasets
- A description of any restrictions on data availability
- For clinical datasets or third party data, please ensure that the statement adheres to our policy

Data are available in the Figshare public data (link in the manuscript).

Human research participants

Policy information about studies involving human research participants and Sex and Gender in Research.

Reporting on sex and gender	*Use the terms sex (biological attribute) and gender (shaped by social and cultural circumstances) carefully in order to avoid confusing both terms. Indicate if findings apply to only one sex or gender; describe whether sex and gender were considered in study design whether sex and/or gender was determined based on self-reporting or assigned and methods used. Provide in the source data disaggregated sex and gender data where this information has been collected, and consent has been obtained for sharing of individual-level data; provide overall numbers in this Reporting Summary. Please state if this information has not been collected. Report sex- and gender-based analyses where performed, justify reasons for lack of sex- and gender-based analysis.*
Population characteristics	*Describe the covariate-relevant population characteristics of the human research participants (e.g. age, genotypic information, past and current diagnosis and treatment categories). If you filled out the behavioural & social sciences study design questions and have nothing to add here, write "See above."*
Recruitment	*Describe how participants were recruited. Outline any potential self-selection bias or other biases that may be present and how these are likely to impact results.*
Ethics oversight	*Identify the organization(s) that approved the study protocol.*

Note that full information on the approval of the study protocol must also be provided in the manuscript.

Field-specific reporting

Please select the one below that is the best fit for your research. If you are not sure, read the appropriate sections before making your selection.

☐ Life sciences ☐ Behavioural & social sciences ☒ Ecological, evolutionary & environmental sciences

For a reference copy of the document with all sections, see nature.com/documents/nr-reporting-summary-flat.pdf

Ecological, evolutionary & environmental sciences study design

All studies must disclose on these points even when the disclosure is negative.

Study description	To examine the ability of individuals to balance the energetic constraints of molting on land we investigate the stomach temperature and movement patterns of molting female SES.
Research sample	A group of female southern elephant seals during the molt, in different colonies of Kerguelen Archipelago: Pointe Suzanne (49°26'S, 70°26'E) between 2014 and 2019, Estacade (49°16'S, 70°32'E) in 2020 and 2022, and Port-Aux-Français (49°34'S, 70°21'E) in 2021.
Sampling strategy	n=55 equipped individuals including:<br>39 with stomach telperature pills<br>18 weighted before and after monitoring<br>The sampling size depends on the year, the movement of the animals during the molt, and how far they come back from the shelter for logistical reasons.
Data collection	Data were collected by field-work volunteers in january-february each year.
Timing and spatial scale	Data were collected during the austral summer molt (December-February) between 2014 and 2022. The field work took place at three colonies within the Kerguelen Archipelago: Pointe Suzanne (49°26'S, 70°26'E) between 2014 and 2019, Estacade (49°16'S, 70° 32'E) in 2020 and 2022, and Port-Aux-Français (49°34'S, 70°21'E) in 2021.
Data exclusions	For stomach temperature pills: we excluded animals with less than 2 days of recording. We also excluded data collected before 6 hours post equipment to discard the effect of anesthesia. Animals far from the shelter were not weighted due to logistical reasons.
Reproducibility	All scientific procedures have been made following the same protocole. We used an algorithm to analyse diving patterns and stomach temperatures making the analyses reliable ans reproducible.
Randomization	Not relevant in this study
Blinding	Not relevant in this study

Did the study involve field work? ☒ Yes ☐ No

Field work, collection and transport

Field conditions	Kerguelen Archipelago (january-february)
Location	Pointe Suzanne (49°26'S, 70°26'E) between 2014 and 2019, Estacade (49°16'S, 70°32'E) in 2020 and 2022, and Port-Aux-Français (49° 34'S, 70°21'E) in 2021.
Access & import/export	Access via the French Polar Institute (Institut Polaire Paul Emile VIctor) and the Terres AUstrales et Antarctiques Françaises
Disturbance	Few females were captured each year. We targeted isolated animals to minimize disturbances.

Reporting for specific materials, systems and methods

We require information from authors about some types of materials, experimental systems and methods used in many studies. Here, indicate whether each material, system or method listed is relevant to your study. If you are not sure if a list item applies to your research, read the appropriate section before selecting a response.

Materials & experimental systems

n/a	Involved in the study	
☒	☐	Antibodies
☒	☐	Eukaryotic cell lines
☒	☐	Palaeontology and archaeology
☐	☒	Animals and other organisms
☒	☐	Clinical data
☒	☐	Dual use research of concern

Methods

n/a	Involved in the study	
☒	☐	ChIP-seq
☒	☐	Flow cytometry
☒	☐	MRI-based neuroimaging

Animals and other research organisms

Policy information about studies involving animals; ARRIVE guidelines recommended for reporting animal research, and Sex and Gender in Research

Laboratory animals	Not relevant in this study
Wild animals	Adult female southern elephant seals were captured during the molt and anesthetized using tiletamine and zolazepam. Loggers were deployed directly in the field and animals were released when they woke up.
Reporting on sex	We investigated females only due to field work considerations. Females molt later than males and this fits better with other scientific procedures conducted in the field. Also, this protocole requieres 2 anesthesia close in time, which is safer on females than for males that are heavier.
Field-collected samples	We did not collected samples for this study
Ethics oversight	All scientifi procedures were approved by the Ethics committee of the French Polar Institute

Note that full information on the approval of the study protocol must also be provided in the manuscript.
